# How Do Physicians Frame Medical Information in Talks With Their Patients? An Inductive Microanalysis

**DOI:** 10.1177/10497323231205152

**Published:** 2023-10-23

**Authors:** Julia Menichetti, Pål Gulbrandsen, Anne Marie Landmark, Hanne C. Lie, Jennifer Gerwing

**Affiliations:** 1 Healthcare Services Research Unit (HØKH), 60483 Akershus University Hospital, Lorenskøg, Norway; 2 Institute of Clinical Medicine, 60504 University of Oslo, Lorenskøg, Norway; 3 Department of Medicine, 60481 Nordland Hospital Trust, Bodø, Norway; 4 Faculty of Humanities, Sports and Educational Science, Department of Educational Science, 11310 University of South-Eastern Norway, Kongsberg, Norway; 5 Department of Behavioural Medicine, Institute of Basic Medical Sciences, Faculty of Medicine, 60504 University of Oslo, Lorenskøg, Norway

**Keywords:** medical information sharing, information framing, video-based study, microanalysis

## Abstract

During medical consultations, physicians need to share a substantial amount of information with their patients. *How* this information is framed can be crucial for patient understanding and outcomes, but little is known about the details of how physicians frame information in practice. Using an inductive microanalysis approach in the study of videotaped medical interactions, we aimed to identify the *information frames* (i.e., higher-level ways of organizing and structuring information to reach a particular purpose) and the *information-framing devices* (i.e., any dialogic mechanism used to present information in a particular way that shapes how the patient might perceive and interpret it) physicians use spontaneously and intuitively while sharing information with their patients. We identified 66 different information-framing devices acting within nine information frames conveying: (1) Do we agree that we share this knowledge?, (2) I don’t like where I (or where you are) am going with this, (3) This may be tricky to understand, (4) You may need to think, (5) This is important, (6) This is not important, (7) This comes from me as a doctor, (8) This comes from me as a person, and (9) This is directed to you as a unique person. The kaleidoscope of information-framing devices described in this study reveals the near impossibility for neutrality and objectivity in the information-sharing practice of medical care. It also represents an inductively derived starting point for further research into aspects of physicians’ information-sharing praxis.

## Introduction

Physicians talk at least half the time of a consultation (Ohtaki et al., 2003; Tai-Seale et al., 2007). During those minutes, they share a substantial, often massive, amount of medical information with the patient, constituting an important source of knowledge that patients can use to manage their health. Appropriate information sharing is indeed an essential part of providing safe and effective care (General Medical Council, 2022). However, patients immediately forget 37%–80% of medical information that physicians had provided and remember half incorrectly (Bravo et al., 2010; Kessels, 2003; Laws et al., 2018; Nordfalk et al., 2022; Richard et al., 2017).

While sharing medical information is essential, the communication strategies physicians use to do so are not well known. In the scientific literature, there is a great deal of attention on *what* information to provide and on aids supplementing the physician talk (Watson & McKinstry, 2009). It is less known *how* they can share medical information during the consultation using various communication strategies. A recent scoping review summarized experimental studies on physicians’ information-sharing strategies and found that only 39 articles out of >9000 reported some detail on physicians’ information-sharing strategies (Menichetti et al., 2021). In the systematic review following that study, only 17 of these studies had a low risk of bias. The systematic review also demonstrated that how messages are framed can have a substantial effect on patients’ information recall and subsequent behaviors, if coherent with the communication goals (Lie et al., 2022). For example, communication strategies with the goal of persuading patients and influencing their thinking and behavior (e.g., being directive, providing argumentations, or negatively framing the message) resulted in significant positive improvements in patients’ behaviors. This is not new knowledge: previous studies have demonstrated that the design choices in persuasive health messages can influence persuasiveness, although the type of choice and their cumulative effect do not matter much (O’Keefe & Hoeken, 2021).

The “framing effect” is well known in disciplines outside medicine. In linguistics and philosophy of language, there is a clear distinction between the propositional content of a message (indicating the essential element) and how the speaker has designed, or framed, that content (indicating the function) (Hanks & Hanks, 2015; Searle et al., 1980; Wittgenstein, 2010). Heritage proposed that no description is strictly compelled by the state of affairs it describes (e.g., propositional content) and is inherently selective (Heritage, 1984, p. 150). By implication, speakers construct every description in order to achieve some specific action, goal, or activity there and then. Indeed, Gadamer (2006) referred to statements as *motivated assertions* that never contain the full content of the proposition’s meaning solely within itself. Therefore, the framing effect, or the products of speaker’s selection process evident in the utterance, indicates how the addressee should take the proposition; that is, it shows its illocutionary act and force (Hanks & Hanks, 2015; Searle et al., 1980). In the tradition of conversation analysis, the specific concept of *action formation* embraces this distinction and stresses how interlocutors design their turns in order to be recognized as particular actions (Schegloff, 2007). Interlocutors can, therefore, make use of resources from the language (e.g., word stress, word order, and use of personal pronouns), or also from the body, the environment, or the position in the interaction, fashioning them such that they can achieve and be perceived as specific actions. Marketing psychology, political sciences, and (health) communication sciences supply the most applied and striking examples of this distinction and of the strength of these framing devices on what messages actually achieve. In political sciences, Robert Entman has, for years, appealed for a cross-discipline, universal framework of “framing,” as an effort of “selecting some aspects of a perceived reality and making them more salient in a communicative text” (Entman, 1993). Along these lines, a substantial line of research investigates *equivalence framing effects*, where in front of two logically equivalent messages conveying essentially identical information (i.e., having the same propositional content), a variation in linguistic presentation can generate different results and cause individuals to alter their preferences (Tversky & Kahneman, 1981). Adding such frames typically involves “casting the same information in either a positive or negative light” (Druckman, 2004, p. 671), thus stressing the benefits (a gain frame) or the costs (a loss frame) of the consequences of a message (Rothman & Salovey, 1997). The gain/loss framing effect is one of the many framing devices that work on the illocutionary force of a message. The field of behavioral economics contributes the notion of allocation framing, where self-allocated framing (making the decision for oneself) can be compared to other-allocated framing (making the decision for someone else) (Li et al., 2021).

Speakers cannot say things without giving a specific shape to how they say those things, and how things are said influences how they will be received. In the dynamic arena of conversational interaction, the subtle ways a speaker might phrase information may not be fully intentional but may operate at the preconscious level (Schmidt, 1992). Indeed, Gadamer called speaking “the most deeply self-forgetful action that we as rational human beings perform” (Gadamer, 2006, p. 26). Often automatic, such variations may be based primarily on contextual elements such as interlocutors’ characteristics, closeness and reactions, the setting, or the preceding discourse (Heylighen & Dewaele, 2002). In medical consultations, where the stakes are high and the intrinsic asymmetry gives the physicians’ words a unique power, this naturally unfolding framing process can be deleterious (or beneficial) if not managed well. Physicians can indeed use, for example, subtle formulations that steer patients toward decisions incongruent with patients’ original stances (Landmark et al., 2016). Physicians’ granular framing practice can also influence what patients are going to disclose (Heritage & Robinson, 2011). In the experimental, conversation analytic study of Heritage and Robinson (2011), it was found that physicians using positively charged words while asking for further complaints (i.e., asking “Is there *something*—vs. *anything*—else you want to address in the visit today?”) obtained almost all the times the desired reply from the patients. Finally, different systematic reviews have demonstrated how message design choices like gain/loss framing or tailored visual designs can influence some (but not all) disease prevention behaviors (Jensen et al., 2012; O′ Keefe & Jensen, 2007).

We know very little about what physicians do to frame the medical information they share with patients in naturally occurring, routine practice. The field lacks descriptive studies of actual practice. This is a general gap in the wider patient education field (Halpin et al., 2021). In the previous literature reviews, medical information-sharing strategies that have been tested, such as information structuring or teach back, were indeed selected as general, top-down, common sense strategies (Lie et al., 2021; Menichetti et al., 2021). That is, the strategies were pre-determined and tested, rather than derived from authentic practice. Such normative studies were designed to see whether common sense strategies worked or not. However, this type of studies does not help gain a better understanding of actual practice nor provide indications about the specifics of communication that naturally work. Such an understanding can serve as the foundation for later experimental studies that would test more ecological and specific strategies.

Thus, the present study took a bottom-up approach, identifying the *information frames* and the *information-framing devices* physicians use spontaneously and intuitively while they provide information to patients. With the terminology around “framing,” we mean the following: By *frame*, as a noun, we mean the underlying constructional system or structure that gives shape to a message; as a verb, we mean the act, process, or manner of constructing a message. By *device*, we mean the use of a mechanism designed to serve a special function (from the Merriam-Webster dictionary). Therefore, we will consider *information-framing devices* to be any dialogic mechanism used to present information in a particular way that shapes how the patient might perceive and interpret it, while for *information frames* we mean higher-level ways of organizing and structuring information to make it more meaningful and useful for a particular purpose. We will therefore apply the more literal meaning of the terms rather than their more frequent use within the traditions of sociology and psychology (see the conceptual review of Guenther et al., 2021).

## Method

This is a qualitative, explorative study, focused on exploring the clinical activity of how physicians share medical information with patients. Analysis was primarily based on microanalysis of clinical interaction (MCI). MCI is a method for “recognizing, identifying, and characterizing any communication phenomena in clinical dialogues” (Gerwing et al., 2023). It is an extension of microanalysis of face-to-face dialogue (Bavelas et al., 2017), tailored to the clinical setting. It is a flexible method that can be used to study any clinical phenomena in an inductive, deductive, or mixed way, either with an explorative or hypothesis-testing approach. Key elements of MCI are (i) the focus on observable behaviors in the interaction, (ii) its context-dependent approach to derive the meaning of a behavior, and (iii) a strong orientation to trace and document any analytical choice. MCI also aims to collect a comprehensive collection of observations. However, in this study, we aimed for saturation of the phenomena. As a result of (iii), one of the first outputs of studies using MCI is a coding manual with definitions, analytical choices, and a collection of examples (the coding manual for this study can be provided upon request). Additionally, the analysis was informed by conversation analytic knowledge on turn design and action formation (Stivers & Sidnell, 2012).

### Data Source

We drew from a corpus of 380 videos of physician–patient interactions available for further research and collected in a Norwegian University Hospital between 2007 and 2008. The videos had been collected in the context of a randomized controlled trial testing a communication skills training for hospital physicians (Jensen et al., 2011). The study was approved by the Regional Committee for Medical Research Ethics of South-East Norway (approval no. 1.2009/1415*)*. Participants gave broad written consent for using their data for further research on clinical communication.

For this study, we aimed for interactions that would be particularly rich in information provision and would have included a wide range of manifestations of information-sharing practices. To meet this aim, we selected the consultations that had scored high on shared decision-making, based on the MAPPIN’SDM tool (Kasper et al., 2012; Kienlin et al., 2017). These 30 consultations were equally divided between those performed before and after the intervention and did not differ compared to the others in terms of physicians’ and patients’ characteristics, physicians’ skills, and patient outcomes. One researcher analyzed this subset of 30 videos until data saturation (pre-settled at three consultations not generating any new information), leading to a final sample of 16 consultations. Table 1 provides details about the setting of these consultations and characteristics of physicians and patients.

**Table 1.** Sample Characteristics (*n* = 16).

ID	Length (min)	Physician gender	Patient gender (age)	Setting	Clinical problem; medical options discussed	Visit
1	28	M	F (48)	Gynaecology	Menstrual pain; hysterectomy versus scraping with hormone spiral	PE; follow-up
2	32	M	F (36)	Gynaecology	Pregnancy; caesarean section versus vaginal birth	PE; new
3	16	M	M (82), with daughter	Oncology	Renal tumor; surgery versus wait and see	PE; follow-up
4	13	M	M (33)	Gastroenterology	Gastrointestinal reflux syndrome and hiatus hernia; surgery versus wait and see	No PE; new
5	19	M	M (60)	Gastroenterology	Undefined stomach pain; biopsy versus not	No PE; follow-up; both second language
6	12	M	M (47), with partner	Orthopaedic surgery	Long-term pain in the knee; surgery versus wait and see	PE; new
7	49	M	M (63)	Nephrology	Kidney stones; stone crushing versus surgery	PE; new
8	12	M	F (77), with daughter	Orthopaedic surgery	Hip pain; surgery versus wait and see	PE; follow-up
9	31	M	M (62)	Orthopaedic surgery	Arm numbness; surgery versus live with that	PE; follow-up
10	16	F	M (57)	Orthopaedic surgery	Swollen wrist; surgery versus not	No PE; new
11	25	F	M (16), with parents	Gastroenterology	Crohn's disease; life-long drug treatment versus anything	No PE; follow-up (patient second language)
12	37	M	F (41)	Orthopaedy	Back pain; further examinations needed	PE; follow-up
13	19	F	M (63)	Anaesthesiology	Planned surgery in the urinary tract; anaesthesia modalities	PE; new (doctor second language)
14	18	M	F (75)	Gynaecology	Osteoporosis and vaginal prolapse; continuation with vaginal ring versus surgery	PE; follow-up (doctor second language)
15	17	M	F (37)	Gynaecology	Post-partum haemorrhoids; incision and drainage versus continuation with nitro cream	PE; follow-up
16	33	M	F (75)	Urology	Urinary incontinence; discussed possibility of surgery but need more examinations	PE; new

PE = physical examination; F = female; M = male.

### Analyst and Research Team

Due to research ethics requirements, the video recordings had to be deleted at the end of 2020. Therefore, before deletion, the researchers who performed the analysis (JM and JG) supplemented the verbatim transcript with observable details such as gestures used by physicians and patients, notable prosodic features (e.g., word emphases), pauses, and various visible contextual elements. Then, the researcher (JM) conducted analysis, discussing and debating analytical choices regularly (approximately every week or two weeks) with another researcher (JG). These meetings began during the process of transcription and involved quality checks of the speech and particularly the co-speech actions and continued over the entire span of analysis, up to and including formulating the coding manual. In addition, throughout the research process, JM brought key points of analysis to a microanalysis seminar group, specifically of five researchers from different disciplines (nursing, medical ethics, psychology, and clinical medicine) all working with video analysis in clinical settings: (1) to discuss examples of what was and what was not an “information-sharing episode,” (2) to ensure that the analyst was not overlooking possible ways of designing messages in the information-sharing episode (by presenting randomly selected extracts and asking to the group to list all the information-framing devices they were seeing), and (3) to check on the overall rationale of the approach and consistency of decisions, particularly when the analyst was unsure (by presenting difficult or unsure cases, asking the group for their views, and discussing until agreement). Finally, to obtain additional peer feedback from researchers who were less familiar with the research process, the results of the analysis and of the categorization process were presented two times to two different multidisciplinary research groups to check and eventually refine the final categories (the information frames).

### Data Analysis

**Figure 1.**
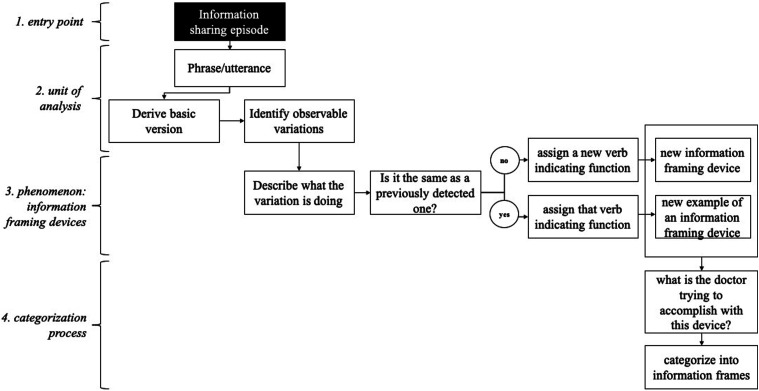
Summary of the analytical process.

The analysis proceeded in four main steps, summarized in Figure 1.

The first step was selecting the episodes when the physician was in an “information-sharing mode,” so that the detailed analysis could focus on the most relevant excerpts. Such *information-sharing episodes* were defined as “physician turns during which *they initiate* sequences of providing *medical information* that is *new to the consultation* and during which *the patient is in a listening role*.” The formal, granular microanalysis was limited to these episodes, and the interactional context was used to interpret and disambiguate the episodes and the behaviors observed.

As a second step, the researcher identified all the various information-framing devices in each information-sharing episode. This included any feature of the message that went beyond a basic expression of the same content; thus, the researcher had to examine each utterance/phrase in the episode to derive the most basic, concise version of its propositional content. For example, if the physician said “this is a VERY big operation,” the most basic propositional content was “this is a big operation.” The researcher then used the basic version as point of reference to reveal the observable variations in how the physician actually conveyed that information to the patient. In our example, the physician added “very” and stressed that word (see further examples in Table 2). The observed features involved behaviors such as prosody, hand movements, and single words to extra phrases and even entire sequences of turns.

**Table 2.** Information Frames and Their Information-Framing Devices, With Examples.

Information frames	Information-framing devices	Example (underscored)	Propositional content from example
*Do we agree that we share this knowledge?*: ensuring that past or present knowledge is actually shared with the patient	Anchoring; Closing; Summarizing; Checking understanding; Marking or exploring shared knowledge; Narrating what is happening	1. *You need to take this drug twice a day, right? I don’t know if I have been clear*… (checking understanding); 2. *you have a size of the uterus that makes it not possible to do the intervention I have talked about* (anchoring)	1. *You need to take this drug twice a day;* 2. *you have a size of the uterus that makes it not possible to do an intervention*
*I don’t like where I am* *(or where you are) going with this*: anticipating or repairing unwanted directions in how the information-sharing process is unfolding	Titrating information; Suspending the utterance; Self-correcting; Correcting; Changing framing device mid-course	1. *You can say that it is ... well it may be obvious to do it* (self-correcting); 2. *the BMI lies quite above … it is under 30 at least* (changing mid-course, in the direction of *softening*)	1. *You can say that it is obvious to do it*; 2. *the BMI lies quite above average*
*This may be tricky to understand*: making complex messages understandable and/or orienting the patient to use an extra effort to fully understand the message	Displaying for the patient to observe; Using verbal analogies/metaphors; Substituting a word with gestures; Specifying; Clarifying; Exemplifying; Explicating; Simplifying	1. *Do not jump into removing the uterus* (using verbal analogies or metaphors); 2. *perimenopause … that period before menopause that lasts maybe one or two years* (clarifying)	1. *Do not remove the uterus*; 2. just using the term *perimenopause*
*You may need to think*: eliciting a reasoning process in the patient	Valuing; Foreshadowing; Providing consequential reasoning; Providing a precis for the information; Sequencing; Contrasting; Comparing	1. *This drug works similarly to Paracetamol* (comparing); 2. *What I want to explain to you is that there are other options* (foreshadowing)	1. *This drug works in this way*; 2. directly saying *there are other options*
*This is something important*: attracting the attention of the patient and directing toward alertness	Accentuating; Signposting priority; Using a double negation; Intensifying; Redirecting; Repeating; Reintroducing; Paraphrasing; Anticipating	1. *This is a very [word stressed] big operation* (accentuating); 2. *the point is that every time you go in and receive surgery so you have the same risks* (signposting priority)	1. *This is a big operation;* 2. *every time that you go in and receive surgery so you have the same risks*
*This is not important now*: presenting the information as something that is routine and does not need special attention from the patient	Minimizing; Deferring; Justifying an action; Framing as certain or obvious	1. *It will be a little bit uncomfortable* (minimizing); 2. *how long we’re going to do this (.) we’ll come back to that* (deferring)	*1. It will be uncomfortable*; 2. *how long we’re going to do this is 2 months*
*This comes from me as a doctor*: expressing or explicating the doctor’s professional role and/or related professional tasks and activities	Using precise terms; Substantiating a medical situation; Showing competence; Declaring the role; Managing responsibility; Being directive; Generalizing; Showing generic practical implications; Filling a pause; Rhetorical questioning; Using a physician colloquialism	1. *This is why I am here, to find the better treatment for you* (declaring the role); 2. *we will use an instrument called resektoskop* (using precise terms)	1. *We’ll find the better treatment for you*; 2. *we will use an instrument*
*This comes from me as a person*: revealing the doctor’s individuality, with the related emotions, reasoning, and vulnerabilities	Providing mixed messages; Displaying own professional reasoning; Relating information to personal feelings; Expressing lack or reduced knowledge; Approximating; Simulating; Subjectivizing	1. *I am wondering if* *we should offer you surgery* (displaying own professional reasoning); 2. *I don’t know what type of genetic you have, almost impossible to say* (expressing lack or reduced knowledge)	1. *We could offer you surgery;* 2. *it is almost impossible to say what type of genetic you have*
*This is directed to you as a unique person*: relating to the patient’s individuality, with the related life circumstances, emotions, experiences, and individual characteristics	Empowering; Validating or relating to the patient experience; Softening; Using humor; Referencing; Making the information explicitly personal for the patient; Individualizing; Translating for the patient; Reassuring	1. *We are doing this so that you can go climbing again* (referencing); 2. *it is also … you get a local anaesthetic* (making the information explicitly personal for the patient)	1. *We are doing this so that you can walk as before*; 2. *it is also ... one gets a local anaesthetic*

The researcher then assigned a label and short definition for each observed information-framing device to capture its function, with the researcher asking: what is this variation doing? For example, adding “VERY” to “big” added a sense of *accentuating* the size of the operation. Since these information-framing devices are rarely made explicit and observable in co-participants’ minimal responses, the analytic procedure was based on the analysts’ knowledge as members of the speech community (Heritage, 1984) rather than on a sequential (next turn) basis. As the researcher moved through the material, she scrutinized each new utterance in the information-sharing episode to detect new information-framing devices and decided whether it could be assigned one of the accumulating list of labels or whether it was distinguishable as a new device. Utterances/phrases could have several devices in place at the same time, and all were extracted. The main purpose of this step of analysis was to extract as many information-framing devices as possible. Thus, variety and exhaustiveness of the different types of framing devices were the aim, rather than establishing reliability and the distribution of devices.

Finally, a fourth step in the analysis was organizing the identified information-framing devices into meaningful categories. This categorization process proceeded in a bottom-up, inductive fashion, where categories were naturally derived from the data. Two researchers (JM and JG) worked together to group the framing devices in categories of broader information frames based on their function. They created post-its with labels for each device and a couple of examples. These were randomly placed on a board, and the two analysts discussed each of them, trying to answer the question: “what may the doctor be trying to accomplish with this framing device?” Information frames were therefore extracted, grouping framing devices with a similar overall function.

## Results

We identified 66 different information-framing devices. These were grouped into nine main information frames conveying: (1) Do we agree that we share this knowledge?, (2) I don’t like where you are or I am going with this, (3) This may be tricky to understand, (4) You may need to think, (5) This is important, (6) This is not important, (7) This comes from me as a doctor, (8) This comes from me as a person, and (9) This is directed to you as a unique person.

Table 2 provides an overview of the nine information frames with their framing devices. A full list of the 66 different information-framing devices with brief definitions and examples is provided in Appendix A. The next section provides an overview of the frames. All examples have been translated from Norwegian into English.

### Attempts at Ensuring Shared Understanding

#### Frame 1: Do We Agree That We Share This Knowledge?

In some cases, physicians *anchored* the message into something mutually known, *summarized* the main information shared, *checked understanding*, *marked or explored shared knowledge*, or *narrated what was happening*, or marked the message as *closed*. All these information-framing devices were used within the frame of checking if the available knowledge so far was indeed shared with the patient. For example, with *checking understanding*, the physicians could conclude an information-giving episode by asking patients whether something was unclear or whether they had questions or doubts. Checking understanding could be done in the form of direct and explicit formulations that offered a clear slot for the patient to confirm or disconfirm understanding (e.g., “Is there something you want to ask me that I haven’t explained?”) or in the form of indirect and implicit formulations that did not provide a slot for the patient to confirm or disconfirm but instead projected a preference for confirmation of understanding from the patient (e.g., “I don’t know what you think is best, but [continued]”).

#### Frame 2: I don’t like where I am (or where you are) going with this

Physicians *corrected* the patient or themselves, *changed framing device mid-course*, *suspended the utterance*, or *titrated information*. These framing devices signaled that the physician was not satisfied with the current state of the message and was responding by adjusting. For example, when *suspending the utterance*, the physician decided not to complete an utterance, signaling that where it was going should be left unsaid and that the patient could imagine how the sentence could be completed (e.g., “and so one has to undergo a big surgery because ... you know”). Another example is when the physicians *self-repaired the framing device*: they started designing the message in a certain way, did not complete, and moved to another way. In these cases, we observed a disfluency in the message: the physician self-interrupted and changed in the direction of a new treatment for how to design the same message. For example, “it takes 5 seconds to check it while we are operating on? you, but if we ... if you are unsure then we don’t do it.” In this case, the physician switched from “if we” to “if you,” thereby changing to a frame that gave more knowledge, power, or responsibility to the patient.

### Orientation to the Information

#### Frame 3: This May Be Tricky to Understand

We observed physicians *clarifying*, *exemplifying*, *explicating*, or *specifying* content, using *visual displays* like pictures or gestures, or adopting *verbal analogies or metaphors*. By mobilizing such extra effort to explain information, the physicians signaled that it might be otherwise difficult to understand and, by implication, that the patient might need to exert extra cognitive effort to fully understand the message. For example, in some cases, the physician selected and presented aspects of the information *using visible modes* of communication, such as turning the screen toward the patient and pointing to it rather than just reading something from the screen. Similarly, one physician did not just describe how a ganglion cyst reacts to being drained but represented the dynamics of its shape and relationship to the patient’s body in gesture. Both these choices allowed the patient to perceive part of the message visually and directly and interpret it along with what the physician was saying.

#### Frame 4: You May Need to Think

Some framing devices oriented the patient to a reasoning process. We observed physicians *comparing*, *contrasting*, *providing consequential reasoning* (“if...then”), *highlighting* and *valuing one message compared to another*, or giving ideas about *the number of bits of information* on the table or *their sequence*. With all these devices, the information frame conveyed was that the patient should think about the information. For example, physicians signaled that the patient should put information in context with other related options when they presented two ideas or contents together to highlight their differences (*contrasting*) (e.g., “the advantage of this last option (.) it’s that you don’t have to be stressed, compared to the first option”) or similarities (*comparing*) (e.g., “Naproxen is a bit on the same street as Voltaren then, so it works as an anti-inflammatory”).

#### Frame 5: This Is Important

We observed physicians *accentuating*, *signposting*, *using a double negation*, *intensifying*, *redirecting*, *repeating*, *paraphrasing*, *anticipating*, or *reintroducing* the message. These framing devices upgraded the importance of a message and directed the patient to be alert and pay extra attention to the information. As an example, with the *accentuating* device, the doctor used terms or formulations that exaggerated or amplified parts of the information without necessarily changing its propositional content, thereby lifting some information into the foreground, so that it emerged more saliently to the patient. This device could take the shape of amplifying adjectives (e.g., not just “a big surgery” but “a very big surgery”), of the use of a stronger expression than what was necessary for utterance comprehension (e.g., not just “a person who will experience chronic pain” but “a person who will suffer from constant chronic pain for the rest of her life”). *Accentuating* could also be a word stressed by using a higher tone of voice or talking slowly while pronouncing a word, or a pointing to some parts of a sentence, just to stress it, like when saying “because I don’t find any clear explanation for what you have (.)” [patient: “no”], and then adding again “I don’t.”

#### Frame 6: This Is Not Important Right Now

At the opposite, physicians designed the message to downgrade its importance. The information was presented as routine and not needing the patient’s special attention or worry. This information frame was done with the devices of *minimizing*, *deferring*, *justifying an action*, or *framing the message as certain or obvious*. For example, when using the *deferring* device, the doctor postponed and deferred a discussion to a future time, thus signaling to the patient that there was no need to pay too much attention to that message at that moment (e.g., “you may need this therapy for a long time, I would say many years, but it depends on how things will go. So how long exactly you are going to take it, we’ll discuss it later”).

### Relationship to the Information

#### Frame 7: This Comes From Me as a Doctor

Some ways of designing the information demonstrated an effort to convey the physician’s professional stance, role, and position. We observed physicians framing the information by using very *precise*, *technical terms*, using the physician *colloquialism*, *substantiating a medical decision*, showing and *expressing competence*, clearly *declaring the professional role*, being *directive*, deciding *who has the responsibility* for an action, *generalizing to the tendencies of the population*, showing *generic practical implications*, or using *ways to keep the turn*. All these framing devices signaled that physicians were stating that message from the position of their professional role. The most explicit example was when physicians *declared their role*, thus defining their professional function, expertise, or tasks. For example, an anesthesiologist talking to a patient about what would happen with anesthesia during surgery said, “So you shouldn’t feel that when we are then working with the urinary tract, [patient: ‘ok’] because it is very uncomfortable. (.) [patient: ‘yes’] So that’s why I’m here” [laughter]. In another case, the physician declared the professional role of another person, giving indication of the borders of professional competency, role, or expertise. For example, one physician said, “and then I’m going to fill the bladder with 300 mL of saline, [patient: ‘mhm’] and then you’re going to cough (.) Then we’ll see how much you leak when you cough (.) [patient: ‘mhm’] And then my boss takes over completely and runs a kind of test on you, a little physical activity.”

#### Frame 8: This Comes From Me as a Person

Other framing devices demonstrated that physicians were talking from their personal position, as a unique person with limitations, vulnerabilities, knowledge gaps, uncertainties, emotions, individual reasoning processes, and doubts. Such information-framing devices were providing *mixed*, *ambiguous messages*, *displaying their own reasoning*, *relating to feelings or emotions*, *expressing lack*, *reduced*, *or approximated knowledge*, or showing that *the information is subjective* and can change from person to person. A frequent example was when the physician inserted the professional self into the reasoning about the present situation, by using a 1st person personal pronoun, followed by verbs indicating a cognitive reflection (I think, I try to understand, I try to clarify, I believe). With this *displaying own reasoning* device, physicians offered a glimpse into their mind and personalized the relationship to the information, thus providing a sort of personal accountability and transparency for deliberations expressed in the dialogue.

#### Frame 9: This Is Directed to You as a Unique Person

Finally, a group of information-framing devices acted within the frame of recognizing the individual person in the patient role, with their own experiences, life circumstances, individual characteristics, preferences, and unique situations. Framing devices used by physicians to signal a recognition that the patient is a unique person involved *empowering* or *reassuring* the patient, *validating their experience*, *softening the message*, *using humor*, referring to *something in the patient’s life*, making *the information explicitly personal for the patient*, *individualizing*, *or translating the information for the patient*. As an example, one physician said, “We’ll fix this [your knee] so you can go climbing on the mountain again.” In this case, the physician was *referencing*, that is, specifically referring to that person’s specific passion for climbing mountains.

## Discussion

In this study, we analyzed video recordings of hospital interactions to identify the specifics of how physicians frame the medical information they share with patients. We identified 66 *information-framing devices* covering nine main information frames, with the intent to ensure shared understanding, to provide orientation to the information, or to display the relationship to the information. To the best of our knowledge, this is the first study that discerned the details of physicians’ information-framing practice inductively in naturally occurring, routine care.

A more overarching finding is that information sharing is not “just” about accomplishing the task of making sure the patient correctly receives the information, but it serves different broad communication goals. We found framing devices and frames that covered task goals of shared understanding and orienting about how to treat the information, but also identity and relationship goals. This reflects the multiple goals theory of communication, where high-quality communication is the simultaneous achievement of (1) task goals (completing a task), (2) relational goals (maintaining healthy relationships with others), and (3) identity goals (managing self-presentation) (Caughlin, 2010; Van Scoy et al., 2017).

The findings of this study also provided details about physicians’ information-sharing framing praxis. Other studies have summarized or tested higher-level, pre-defined communication strategies (Lie et al., 2022; Menichetti et al., 2021). Some of the information-framing devices identified in this study overlapped with these previously tested communication strategies (e.g., repeating, simplifying, checking understanding, and expressing lack or reduced knowledge) (Menichetti et al., 2021). However, with this inductive study, the level of detail was much higher (e.g., we were able to discern functional differences between *repeating* and *reintroducing* information). Some of the framing devices also resembled Wittgenstein’s examples of language games, like describing an object by its appearance or its measurements, speculating about the event, or forming and testing a hypothesis (Wittgenstein, 2010). These language games display, indeed, the goal or function of linguistic expressions as part of an activity or a form of life (Wittgenstein, 2010). In this way, language games are more similar to information-framing devices in their variety, but perhaps less granular. We believe that the granular approach to information-sharing praxis here provided may offer an ecologically grounded foundation (i.e., close to the actual practice of physicians in their natural, work environment) for designing future studies aimed at testing specific information-framing practices.

Other previous studies have also focused on one specific information-framing device in order to disentangle its layers of functions (Stevanovic, 2013). As an example, *displaying own professional reasoning*, for example, including “I’m wondering if …” as part of a proposal (see Table 2), has been previously studied in its different functions (Stevanovic, 2013). This device can indeed also function as a resource to give more decisional or moral, deontic responsibility and obligation to the other, or reducing the asymmetry, as shown in Stevanovic (2013). Further layers of functions of the devices and information frames could, therefore, exist and may warrant further exploration. Other studies have also focused on the interactional achievements or patient outcomes related to one specific information-framing device (Delli et al., 2022; McCabe et al., 2013; Peräkylä, 2002). For example, McCabe and colleagues (2013) found that the repairing practice of patients and psychiatrists (which included what here we labelled as *self-correcting*, *correcting*, *changing framing device mid-course*, and *checking understanding)* influenced patients’ adherence 6 months later. Similarly, Peräkylä (2002) found that physicians used the device of *substantiating a medical situation* when delivering diagnostic information in circumstances when they are dealing with possible resistance by patients, and most importantly, this device can actually encourage the patients to talk. With this study, we therefore provided a sort of kaleidoscope of information-framing devices, which can constitute an inductively derived starting point for further research into aspects of information sharing (or “tell” moments). Each information-framing device can indeed be a starting point for further in-depth exploration. Further research can, for example, identify the different layers of functions of the framing devices, but also describe how information-framing devices are taken up by interlocutors and where or under which circumstances they occur. Previous literature has demonstrated that how messages are framed can greatly influence how listeners perceive the message, thereby shaping their attitudes and opinions (Scheufele & Tewksbury, 2007). Even a single word can change how many concerns patients present to the physician (Heritage & Robinson, 2011). Thus, further research could particularly explore whether some devices are more successful than others in terms of patients’ understanding, recall, or even adherence when the message touches on decisions and plans for after the consultation.

In this study, we defined information-framing devices by first articulating the most basic, propositional content for the utterance under scrutiny. This approach was based on well-grounded assumptions that there would be observable differences between the basic content and the way the physician stated the information. We observed that there was no instance when a physician’s utterance matched the mere “propositional content.” Consider the following example:

1 D: Yes, it is.
2 P: Yes (.)
3 D: And it’s … it’s not harmful to take those. But do you have a lot of
4 trouble that you bleed very easily or get bruises?
5 P: No, I don’t think I bleed easily, because you see … I don’t bleed now after taking [unclear].
6 D: Then you can continue with it.
7 P: Okay.

In line 7, the physician used an approach as closest as possible to the propositional content “you can continue with it,” but by adding “then,” he inserted a *providing consequential reasoning* framing device. The observation that physicians always shaped information in their utterances reveals a near impossibility for pure neutrality and objectivity in the information-provision practice of medical care (as in any information-provision activity, see Entman, 1993). When one takes a photograph, there is always an aspect of selection and interpretation. One frames the subject in different ways—zooming in or out, situating the focal point in different parts of the frame, and using shadow, light, contrast, and focus to bring attention to different aspects of your subject. So just as a photographer can never present a subject “neutrally,” no physician can provide information to a patient neutrally. This may have implications for some of the (contested) ideals or principles proposed to medical practice, like impartiality, neutrality, or objectivity in information provision and shared decision-making (Hoehner, 2006; Orentlicher, 1992). Wittgenstein even warned against valuing the close correspondence between proposition and expression with the term, suggesting that *exact correspondence* (which resonates with notions of impartiality, neutrality, and objectivity) carries a sense of praise, whereas inexact a sense of reproach (Wittgenstein, 2010). A narrative review concluded that information sharing in the context of “shared decision-making is fraught with risks of cognitive biases and undue influence of even the best-intentioned physicians and family members” (Ozdemir & Finkelstein, 2018, p. 6). These authors concluded by advocating for efforts (e.g., decision aids, de-biasing strategies, and inclusion of third parties) to minimize these influences and reduce the power distance that amplifies them. We similarly conclude that, first, one possible remedy can come from raising awareness of the variety of framing devices physicians use in authentic (medical) conversations with patients and better equipping them to construct message. The extracted list of framing devices can thus serve as a foundation for teaching modules for practitioners that can orient them toward their framing behaviors, teaching them to be aware of what they are actually doing, what effect their framing devices could have, and whether those effects are consistent with their intentions. Since information-framing devices can be used intentionally or unintentionally, a greater understanding of these devices can help to avoid unintentional biases or misunderstandings. A similar conclusion was raised by Entman 30 years ago, when discussing the inevitability of framing and the tendency of objective reporting in journalism practice (Entman, 1993). A second remedy can be a greater focus on asking, listening, and understanding patient stances so that the framing devices in use would incorporate and fine tune with the patient at the highest grade possible. Indeed, the taxonomy of natural information-framing devices physicians use to share medical information with patients can be seen as a deepening into the “tell” moment in the interactional “ask, tell, ask” process. In that “tell” moment, sharing information appears as always positioned and conveyed in fine-tuned, tailored ways to achieve more global and specific goals (e.g., securing understanding and alignment), which can be more or less aligned with patient stances and goals collected by listening carefully to patients after the initial “ask.”

Some of the limitations of this study include the use of transcripts supplemented with additional notes for the analysis. Therefore, although we included visible actions that were included in the transcripts, the analysis focused primarily on speech. Another major limitation in terms of reliability of findings is the fact that the analysis was mostly performed by one researcher. Hence, we cannot assume that two different researchers would have identified and labelled all the same data segments independently and similarly. This approach precluded creating a reliable coding system; however, creating such a system was not the goal of the study. Similarly, since the study focused on the breadth of information-framing practice, we did not provide an exhaustive collection of examples nor did analyze the entire material. Lastly, although sense-making is “strongly interactive and contextual” and context sensitivity is a universal property of communicative practices (Linell, 2009, p. 13), the analysis took a limited approach to incorporating context. Specifically, the overall context of the medical consultation informed the purpose, and the sequential unfolding of the dialogue informed decisions as to where information-sharing episodes occurred; however, the granular level of analysis was somewhat decontextualized from the interactions as a whole. The analysis presented here necessarily focused strictly on those sequences during which the physician is actively sharing information with a patient who is providing signals that the physician can go on (i.e., using *continuers* such as nodding or saying “m-hm”). This also means that, due to the focus of this study, patients and their contributions were more or less invisible. However, the conversations constituted continuous opportunities for intersubjective understanding and misunderstandings, occurring in and outside these particular episodes. As with all interlocutors, for physicians and patients, intersubjectivity is a practical problem routinely solved and achieved (Schutz, in Heritage, 1984, p. 54). Follow-up work constructed from the results presented here would benefit from incorporating the patients’ roles more specifically, including how their actions in the dialogues elicit how physicians frame information and how they respond to various types of information-framing devices.

## Supplemental Material

Supplemental Material - How Do Physicians Frame Medical Information in Talks With Their Patients? An Inductive Microanalysis Click here for additional data file. Supplemental Material for How Do Physicians Frame Medical Information in Talks With Their Patients? An Inductive Microanalysis by Julia Menichetti, Pål Gulbrandsen, Anne Marie Landmark, Hanne C. Lie, and Jennifer Gerwing in Qualitative Health Research
