# Supplementary material for: How Do Physicians Frame Medical Information in Talks With Their Patients? An Inductive Microanalysis
Source: Qual Health Res. 2023 Oct 23;34(1-2):101–13. doi: 10.1177/10497323231205152 (PMC10714701; doi:10.1177/10497323231205152)
Supplement: Supplemental Material - How Do Physicians Frame Medical Information in Talks With Their Patients? An Inductive Microanalysis [file sj-pdf-1-qhr-10.1177_10497323231205152.pdf]

Appendix A

The following pages summarize the 66 extracted information framing devices, organized based on the nine main information frames, with a brief explanation and a selection of two or more actual examples each, translated from Norwegian to English.

1. **Do we agree that we share this knowledge?**

Anchoring: the doctor secures new information into something mutually known from the patient's past or earlier moments in the dialogue. This framing device shows how the doctor is integrating the new information into what they have discussed, giving the patient the opportunity to contextualize it that way as well.

a. Not just ”you have a size of the uterus that makes it not possible to do an intervention”, but ” you have a size of the uterus that makes it not possible to do the intervention I have talked about” (referring to previous moments in the dialogue)
b. Not just “you seem well” but “Doc: Now...you seem much better than the last time Pat: Yes, it cannot be compared. My God [unclear] then I was lying like that [showing with body]

Closing: The doctor finishes a message by adding a statement that indicates that this message is irreversible.

a. ”it's one of two either to just wait and see, (.) [pat "yes"] or to intervene. That's the way it is.”
b. ”then you should know that it is not a major operation, but you have a small risk of inflammation and such and such, that is it, but it is not a major operation. [pat "exactly”]

Summarizing: The doctor provides a concise overview of the information, by summing the key information messages already conveyed (differently from the formulation of providing a precis of the information, which is used to sum up and anticipate the number of information bits). This device is usually (but not necessarily) used at the end of a more detailed sequence of messages, and allows the patient to have an overview of the information she/he has received in an information giving sequence. This framing device overlaps with *reintroducing*, as the contents of the messages are reintroduced later on and summarized in a concise way; however differently from *reintroducing*, we see with this framing device a long turn with a list of information messages already provided, and the doctor is explicit about the intention to provide a concise summary as from the use of some verbal signs e.g. “and so/then...”, “so what I suggest is...”.

a. But I suggest this... you use these two, and then you take blood tests every two weeks at your GP, (.) [mother "yes"] and then I'll set you up here again for a check-up in 3 months. (.) I will set you up, when it will be... it may not be 3 months, but it will be... I will set you up so that we do not lose contact, so that we only hear how things are going with both Imurel and you and all that. [look at the patient] And then I will try to talk to some adults about how long we will have all those drugs. But regardless, the plan is that you will use this for a long time to come. (360, 167)
b. “Then we leave it there. Then I won't do any more of it today. Then you will get your knife back. No, then. You are fine, and why treat anything further then. Then I will not set you up for any new control.”

Checking patient's understanding: The doctor concludes an information giving episode by asking the patient if something is unclear or if he/she has questions or doubts. With this framing device, the doctor not only gives the patient the opportunity to raise concerns, doubts, and needs, but also to ask clarifications or explanations for unclear messages or passages. *Checking understanding* can be in the form of direct and explicit formulations that offer a clear slot for the patient to confirm or disconfirm understanding:

a. "Is there something you want to ask me that I haven't explained?"
b. "Yes any..do you have any questions or..."
c. "It is the uterus which has this shape, do you see it?"

Checking understanding can be also in the form of indirect and implicit formulations that don't provide a slot for the patient to confirm or disconfirm but instead projects eventually a preference for confirmation of understanding from the patient, like:

d. And secondly, I make sure in a way that the samples I request are caught [smile], [pat "mhm"] right. [pat "mhm"] I don't know what you think is best, but [continued]
e. With the urodynamic examination, you find out how correct it is to operate on you. [pat "yes exactly"] After the examination, they can sit and discuss with you that, for example, you have such a great (.) chance of getting better (.) right?

Marking or exploring shared knowledge: The doctor checks with the patient that they share the same knowledge, assuming that the patient does have the knowledge that doctor wants/expects them to have. This framing device in its "marker" stance is usually expressed as "you know", indicating that the doctor is opening for agreement from the patient that they share the same knowledge (first example). The framing device can also be used with a more explorative stance, when the doctor questions the patient directly about his or her level of knowledge on a specific topic, offering the opportunity to fine tune the type and amount of information that needs to be provided (second example).

a. No, so I would [lying back on the chair] recommend it to people if they have problems from it. Because if they don't have problems or think it's cosmetic. But you also understand that there are a lot of people who think this has been troublesome, you know.
b. Then, in addition, in recent years, butox has appeared - you know that in rakfisk and butolin [both laugh] have you heard about it?

Narrating what is happening: The doctor introduces future or describes his/her present actions using temporal pronouns that anchor the information to the current/soon to happen situation. This framing device is usually used just before or during the physical examination. Note that *narrating* is often intermingled with imperatives to the patient regarding what they should be doing when. These imperatives are excluded as part of this formulation because the doctor will immediately have direct evidence of patient understanding (i.e., whether or not the patient does the behaviour), e.g.: "So. Now we take a small break, now you can breathe again".

a. Now the worst is soon over. Right, now I'm leading this pipe through. Just have to lie down heavily with your buttocks and then breathe with your stomach. And then we use a little water to... When we get to the sphincter around here, it's especially important that you breathe heavily and let your bottom hang down. Totally heavy in the ass. So it's a bit uncomfortable right now.

2. **I don't like where I or you are going with this**

Suspending the sentence: The doctor does not complete an utterance, which therefore lacks key elements that would be required to grasp its meaning. The concept expressed in the sentence appears suspended, floating, truncated, so that it is lived at the patient and his/her imagination how the sentence will possibly conclude. The doctor thus signals that something is left unsaid and the patient has the ability or the knowledge to imagine how the sentence can be completed, which is sometime marked more explicitly with the term "ikke sant" (you know).

a. "This is a surgery in anesthesia. So...." [nudging towards an implication]
b. "and so one has to undergo a big surgery because...you know. [nudging towards a reason]

Titrating information: A sequence of turns where the doctor provides small pieces of information, each projecting sufficiency, but due to patient's prompts, follow ups, requests, or contributions, continuing to provide further pieces of information. This framing device gives a sense of providing information almost reluctantly, at the demand of the patient. At every bit, the doctor provides signals that more explanations are not needed, with generalizations or efforts to refer back to what the patient already knows. This framing device is positioned at a macro pattern level about how the sequence unfolds.

a. 51 Doctor Has it been a long time since you... Were you hospitalized in (.) abroad recently?
   52 Patient No, it was 3 years ago.
   53 Doctor No, because if you become one, there are strict quarantine rules and such.
   54 Patient Yes, I'm aware of that, although I don't quite understand why, but fine.
   55 Doctor No, they have got the kind of bacteria that we don't want [PT "YES YES OK"] to have. You've probably heard it before.
   56 Patient Yes, I am aware of that. But they've been very... the hospital I've been to in Spain it's been absolutely fantastic.
   57 Doctor Yes. No, that has nothing to do with it.
   58 Patient No, I guess it's just safety... I haven't been to a hospital there for 3 years, so...
   59 Doctor There are different bacteria in Europe in general than there are here. hmm
   60 Patient Yes. I am very nervous about the test that I am going through now. So just tell me if I seem like that, yes

Correcting: The doctor explicitly marks or points out an error in the patient's previous utterance, by marking an error or negating what the patient said.

a. P: "afterwards it was [unclear] with you"
   D: "<u>No</u>, it was probably another colleague, but it doesn't matter".
b. P: "Yes, I am aware of that. But they've been very... the hospital I've been to in Spain it's been absolutely fantastic".
   D: "Yes. <u>No, that has nothing to do with it"</u>.

Self-correcting: The doctor recognizes and repairs an error in the initiated utterance. Differently from *changing formulation mid-course,* there is some mark of error (e.g. by negating or contradicting what said).

a. "[continued] And when you have to measure a baby that is inside the belly, it is very important that you get to the right level. If you imagine you're going to measure the circumference of a salami sausage, you have the salami sausage here, and then when you do [unclear] you sort of cut yourself... or don't cut then, but you know what I mean, right through there. And if you do it at an angle, then you might get the impression that the baby is bigger than… you know what I mean?"
b. "[continued] Because, as I said, it will twist a bit, so that we get these very tight, these bad, bad pains that are very similar... or not very similar, but they can resemble... because they come like that... [continued]"

Changing formulation mid-course: The doctor starts framing the message in a certain way, does not conclude and moves to another way. In this way, we observe a disfluency in the speech, where the doctor interrupts and changes approach in the direction of a new treatment for how to frame the message. These cases show online monitoring of how the patient might receive the message and thus indicates a deliberate effort of making the information palatable and a strategic use of a formulation (which becomes an information sharing "strategy"). Therefore, this results as a meta-level device, where we see both a *changing formulation mid-course* and a specific information framing device (the second, newly framed one). In both examples below, the change from one framing device to another is marked by an ellipsis (…).

a. Oh no. And that's why I... we know very well (.) that exactly you have been very difficult for us, (.) [formulation changed from "I" to "we", in the direction of *managing responsibility*]
b. The BMI lies quite above…it is under 30 at least. (.) [formulation changed from "above" to "under", in the direction of *softening*]

3. **This may be tricky to understand**

Displaying for the patient to observe: the doctor selects, presents and augments aspects of the information using visible modes of communication simultaneously with the speech. This framing device makes the information observable to the patient, so the patient can perceive it directly.

a. Not just saying «stomach», but pointing to the stomach
b. Not just describing how a ganglion cyst reacts to being drained, but representing the dynamics of its shape and relationship to the patient's body in gesture
c. Note just reading something from the screen, but turning the screen and pointing to it to allow the patient to view it

Using verbal analogies or metaphors: The doctor presents aspects of the information using imagistic speech like rhetorical figures. This device makes the abstract idea behind an information more concrete, by using knowledge of concrete concepts that resemble the abstract idea.

a. "Don't jump into removing the uterus as first choice"
b. When referring to information: "You can digest this...you have time to digest this"

Substituting a word with gestures: The doctor uses a *displaying for the patient to observe* device (i.e. the doctor selects and presents aspects of the information using visible modes of communication) in place of speech. Therefore, this is not a *suspending the utterance* device, as the doctor completes the sentence but by using other communication modes.

a. “if you poke a hole in it then [claps the hands] [Pat “yes”] And then it comes again. And then it comes again(.)”
b. “Because if you have an ulcer in the anal canal, it leads to a spasm in the sphincter muscle. [opens a closed hand] The cramp is almost like if I take my hand... [shows the hand closed] you see now it's well soaked here, [pat "mhm"] so you can ... if you imagine that there is a wound here and this is the anal area, then it leads... when you have a bowel movement, stool with an acidic pH comes down into the wound – [pat "mhm"] cramp(.) Then see you it gets animated or white around here. [points to the hand closed] (.)

Specifying: The doctor moves from general to specific information, potentially reducing patient cognitive effort by gradually narrowing the focus: the general part orients the patient to a domain, then the specific part identifies the relevant part of that domain precisely.

a. Not just ”chronic pain”, but ”pain…chronic pain in the abdominal area at the bottom”
b. Not just ”this surgery that we do (.) is associated with adhesions”, but ”this surgery that we do (.) especially in the pelvis is associated with adhesions”

Clarifying: The doctor links a technical term, definition or explanation to a lay term, definition or explanation; (both) should refer to the same thing. This information framing device addresses the potential for a lack of shared understanding of terminology so that the patient can know the meaning of a medical term/definition/explanation, both in how the doctor is currently using it and when the patient comes into contact with it later.

a. Not just using the term “Perimenopause” or using the definition in an utterance, but linking the two together: “Perimenopause.. That period before menopause that lasts maybe one or two years”
b. Not just “A small uterus” but ”And this is...(.)ca. 5 cm, 50 mm as diameter at the thickest. A small uterus (.)”

Exemplifying: Providing concrete examples that illustrate the information message in a specific situation or context.

a. **“**And in situations where, for example, you have to go down on your knees or as you have experienced... a little extra, [pat “yes”] then it slides into the joint.”
b. “With the urodynamic examination, you find out how correct it is to operate on you. [pat "yes exactly"] After the examination, they can sit and discuss with you that, for example, you have such a great (.) chance of getting better (.) right.”

Explicating: The doctor makes explicit an implicit term, usually disambiguating a pronoun or a pause/silence. [Note that *explicating* is different from *specifying*, as the information is already provided implicitly and the doctor does not add new information].

a. “No you don’t feel it (.) you don’t feel any pain”

Simplifying: Providing information in a way that includes extremely general and lay terms (e.g. “thing”, “something”). *Simplifying* seems to orient the patient that the exact terminology is not necessary to understand the main message.

a. “Because now we have examined you with so many different tests and examinations (.) but things are not as they should be”.
b. “there is only that the radiologist has written something”

4. **You may need to think**

Valuing: The doctor provides the pros or cons only for one option but not the other. In this way, one option stands out as preferable or more valued by the doctor.

a. Another option is that I set you up for a fixed check. (.) [pat: "mm"] The advantage of the latter (.) [PT "mm"]is that you don't have to fuss, first of all. And secondly, I make sure in a way that the samples I request are caught [SMILE], [pat: "mm"] not true.
b. If I am in doubt (.) then I am going to apply [closed arms] you for another examination called urodynamic examination, a more advanced one than what we have here with various - not painful, no -(.) [pat: "NO"] with various catheters that measure pressure in the urinary bladder and urethra [pat: "yes well"] and the vagina and...

Foreshadowing: the doctor signals that new information is coming. This framing device allows the patient to prepare for an information giving sequence. It is different from *summarizing* in the sense that the information provided is new to the interaction and the device shows an effort of preparing the patient for an information message coming.

a. Introducing an information sequence with e.g. “What I want to explain to you is…” (foreshadowing that new information is coming)
b. “Good (.) then I think we can conclude that your diagnosis has been that you had a fissure (.) It was a very difficult examination because there is such infamous pain with a fissure.” (foreshadowing that a conclusion is coming)

Providing a precis for the information: The doctor provides a concise overview of the information, by summing the number of the key information messages. This information framing device is usually used at the beginning of a more detailed sequence of messages and allows the patient to have an overview of how many bits of information she/he is going to receive in an information giving sequence.

a. ”And there are always ((two possible explanations)) [sign of ”two” with fingers of the hand] (.) [pat "mm"] for this (.)”
b. ”and so there are two types of c-sections. One...”

Providing consequential reasoning: The doctor embeds new information in a consequential structure so that the patient can understand the precursors and consequences (and points of uncertainty). This framing device draws the patient’s attention to branching, hypothetical futures that are natural consequences of decisions/actions/outcomes so that the patient can grasp potential contrasting implications and how the information fits into a broader, contingent structure.

a. IF/THEN formulations (e.g. “If one gets adhesions then one gets a bigger change of having pain...”)
b. SO/THAT formulations (e.g. “You have had polyps on the cervix, [PT "mmm"] and so there are greater chances that you may have polyps in the uterus (.)”)

Contrasting: The doctor associates two ideas or contents highlighting the differences between them. With this formulation, the state of being strikingly different from something else in juxtaposition or close association is stressed. This framing device is usually manifested by the pronoun “but”.

a. “it means that you are going to be operated at the X hospital (.) but you will receive indications about what to do and what is going to happen and everything else here”
b. “the advantage of this last option (.) [P: "mhm"] it is that you don’t have to be stressed, compared to the first option”.

Sequencing: The doctor organizes the sequence of information messages by using verbal signs that divide the messages in macro-units of meaning and forecast that other messages are (or are not) going to arrive after the first. This information framing device is easily complemented with gestures that augment verbal signs of ordering with visible modes.

a. ”And the one [sign ”one” with the finger] is that something must have happened to that muscle anyway and that they haven't discovered it. But the other [sign ”two” with fingers] that we occasionally see, (.) it is that [continue]”
b. ”And then there are two types of caesarean section. There are those that you already know about when the baby is made, and then there are those that you know about (.) an hour before it is made. [Unclear] And then you come with such an intermediate (.) group, and then we have a somewhat different solution.” [also *providing a precis of the information*]

Comparing: The doctor associates two ideas or contents highlighting the similarities between them. With this formulation, the state of being similar to something else in close association is stressed. This framing device is usually manifested by formulas like “the same as”.

a. Oh yes. mmm.(.) Because Naproxen is a bit on the same street as Voltaren then, [P: "YES"] so it works as an anti-inflammatory if there is a bit of inflammation in the muscles as well. [P: "YES"] [PAUSE] (277, 98a)
b. And nitroglycerin cream has it. (.) you are I perhaps explained it last time - but the same effect that is used in angina pectoris and chest pain [touches his chest] can be placed under the tongue and... [pat "mhm"] It relaxes the muscles in the heart and blood vessels so that the blood pressure falls and the chest pain or angina goes away. [touches his chest] Here we use it on the anal opening.

5. This is something important

Accentuating: the doctor exaggerates (amplifies, emphasizes) parts of the new information without necessarily changing its propositional content and seems to be designed to enhance the chances that the patient will pay attention to it. The *accentuating* device lifts some information into the foreground, so that it emerges more saliently to the patient.

a. Accentuating can be amplifying adjectives:
    i. Not just an “intervention”, but a “very big intervention”
b. Accentuating can be using a stronger formulation than is necessary for utterance comprehension

ii. Not just ”a person who will experience chronic pain”, but ”a person who will suffer from constant pain for the rest of her life”

c. Accentuating can be stressing a word by using a higher tone of voice or talking slowly while pronouncing a word, thereby drawing attention to it in the context of the utterance.

iii. Not just ”I can’t find your ovarian reserves”, but ”I canNOT (WORD STRESSED) find your ovarian reserves”

d. Accentuating can be pointing to some parts of an utterance, just to stress that part

iv. “Because I can’t find an obviously good explanation for this immediately (.) [PT: "no"] I can’t find it (.)” [Note that this is different from the formulations *repeating* and *reintroducing* because the repeated words do not convey meaning on their own because they are elliptical, with a deictic reference to the earlier utterance.]

Anticipating: The doctor states something that pre-empts something the patient could reasonably ask about, but that the patient hasn’t asked about yet. Therefore, the doctor is making something happen earlier rather than later and without elicitation from the patient, while also displaying what the doctor expects the patient to be interested in.

a. Not just “I don’t know how many seizures you should expect” but instead “Also if you ask me [coughing] if you can expect to have seizures, how many and so on, so I become like a question mark [SIGN QUESTION MARK] Also I cannot answer you” [overlap pat "no"]
b. If I am in doubt (.) then I am going to apply [closed arms] you for another examination called urodynamic examination, a more advanced one than what we have here with various - not painful, no -(.) [pat overlap "no"] with various catheters that measure pressure in the urinary bladder and urethra [pat overlap "yes well"] and the vagina and...[continued talk] [Note that this inception from the doctor is not justified by any sign from the patient, so it results as a –more indirect then the previous - anticipated reply of a possible patient question]

Intensifying: This information framing device can be positioned both at a macro pattern level about how the sequence unfolds or as sequence of words within the same sentence. It involves a sequence (of turns or words) in which the doctor increases the strength of the message incrementally, in a sort of ascendance. When positioned at the macro pattern, the doctor provides bits and pieces of information on the same topic, in an intensifying ascendance.

a. 45b Doctor It's good that it's going well then, isn't it.
45c Doctor But I think we'll just have to decide to... I x...
46 Mother It's no problem, I [unclear] We buy new medicines.
47a Doctor ((The meaning is that he should take it (.) permanently then, [nods] (.) that's it. Because we... it's that... (.)So))
b. [laughing] No, it's fine that you speak up. [PT "yes"] Then we can also talk about it. You don't have to be fully awake and ready and follow everything, [PT "no"] you don't have to.

Paraphrasing: Conveying the same information message twice, by restating the same meaning of a text or passage using other words. With this framing device, the doctor attempts to preserve the essential meaning of the message by formulating it in another way. This framing device differs from *clarifying* as it does not provide an extended definition of a term, but instead a rephrase of the same message in other terms.

a. ”I actually think it is a good solution (.) I think it is a wise choice to take if you do it”
b. ”But... then there is it’s small but it is here back at 6 o'clock – you mentioned a small knot that you felt. I also feel a small knot there, and it is bluish, discolored blue black.”

Redirecting: With this information framing device, the doctor explicitly redirects the attention to a different topic with the use of signposts.

a. P: But I'm missing some discs, I've been doing that for a long time.
D: But (.) now back to that bullet. [PT "YES"] (.) I have seen now, there is no ulceration. You have to be good at using that cream.
P: Yes. (414, 124)
b. If we leave it for a moment. If we go back to it with that sample. I will almost have to find out if we should order such a test or if you think it is a bad idea.

Reintroducing: The doctor comes back to information that has been shared previously in the visit. This happens in another moment of the conversation, with the doctor expressing a proposition that is entailed by another utterance already said in the discourse situation. This framing device has the function to go back to that message.

a. In visit 230:
Turn 300. I haven't seen anything wrong, but I haven't been able to see the whole thing.
Turn 310: I couldn't see anything wrong, but I couldn't see everything in the bladder(.).
Turn 336: But we've now seen most of it. And it looked good.
b. In visit 325:
Turn 33: The operation [WORD STRESSED] is not a big operation.
Turn 53: But as I said, it's not a huge operation.
Turn 67: But as I said, it is a completely normal operation, we operate on many of them and therefore have day surgery.
Turn 131: then you should know that it is not a major operation, but you have a small risk of inflammation and such and such, that is it, but it is not a major operation.

Repeating: The doctor says the same information more than once in the same turn unit, with the doctor using exactly the same words to express the same information message (differing from *paraphrasing* as the wording is exactly the same).

a. Bring something to read, (.) because it could be a long day(.) Something Agatha Christie or something like that, something light. Because it could be a long day.
b. The operation [STRESS ON WORD] is not a big operation - we use to, just so you know what you are saying yes or no to (.) - the operation is not a big operation [while touching the patient arm and looking at it], we use to do it under anesthesia or numb the whole arm [touches all the arm] [P: "mhm"]...

Signposting priority: Highlighting one piece of information by explicitly stating that this is the main piece of information. The information would stand on its own without prioritizing, but the meta-communicative effort of prioritizing foregrounds the information against the background of other information.

a. Not just «every time that you go in and receive surgery so you have the same risks...», but «the point is that every time you go in and receive surgery so you have the same risks...»

b. ”Yes. No but this was fine. The most important thing is to keep yourself as you are now, that you don't relapse, that's the point.”

Using a double negation. Instead of using a more linear affirmation, the doctor uses a formulation that includes a pair of negative terms to express the same message.

a. Not “the age doesn’t affect the possibility of surgery”, but “the age is not a contraindication for not having surgery”
b. Not “there is a small group of patients who continue to have these symptoms”, but “there is a small group of patients who do not get rid of these symptoms”.

6. This is not important now

Deferring: The doctor projects a decision to a future time rather than making it now.

a. “...for a long time(.) I say several years now, just to have said it. Of course, it depends a little on how good you are, etc., etc., but I wouldn't dare take it away now for the next year... a few years to come. [was looking at the father, and now looks at the patient] How long we're going to do this (.) we'll come back to that.”
b. So, therefore, I think I will call you again in a couple of weeks (.) and then I will get hold of a Vietnamese interpreter, and then we can talk better.”

Framing as certain or obvious: The doctor frames what is coming next as certain or obvious, increasing the solidity and certainty of the message. It can be done by using more or less direct formulations. Here are some examples listed from less to more direct formulations:

a. that must of course be taken into account in the calculation.
b. And it is clear that if you have severe seizures, it is a reason for hospitalization.
c. Instead of "And there are two possible explanations for that", saying "And there are always ((two possible explanations)) [PT "mhm"] for that (.)"

Justifying an action (present or future): The doctor includes a statement that goes in the direction of explicating, defending or validating a present or future action.

a. “You will then receive a letter from the department stating that the application has been received - there is a lot of paper to be sent - and then the practical messages in connection with bathing”
b. “Let's see, I'll just fill out some paper and that's it. (.) There are so many diagnostic codes and stuff that need to be dealt with.”

Minimizing: the opposite of *accentuating*, the doctor uses terms or formulations that minimize parts of the new information without necessarily changing its propositional content. This framing device reduces the strength of parts of the information and seems to be designed to present new information as something that is routine and does not need a special attention or worry from the patient.

a. Not just “uncomfortable”, but “a little bit uncomfortable”
b. “And so the only thing is just to wait to be called for the surgery”

7. This comes from me as a doctor

Being directive: Framing the information message as an order or a strong indication. The message is for example framed with the imperative form of the verb, with the use of formula like “don’t”, “you must”, or adding an extra phrase that does not leave the possibility for the patient to act differently without opening a conflict or losing face.

a. There are a number of things associated with surgery, complications with bleeding, infection... right(.) Have it as a last option (.) Trust me.
b. Drink more. [PT "mhm?"] You must drink at least two liters of water a day.

Declaring the role: The doctor declares or defines his/her role and function to the patient. In this way, s/he clarifies the boundaries of his/her expertise and takes responsibility for an area of action.

a. An anesthesiologist talking to a patient about the anesthesia during surgery: “So you shouldn't feel that when we are then working with the urinary tract, [P: "ok"] because it is very uncomfortable. (.) [pat: "yes"] So that's why I'm here.”[laughter]
b. And then I'm going to fill the bladder with 300ml of saline, [pat "mhm"] and then you're going to cough (.) Then we'll see how much you leak when you cough (.)[P: "mhm"] And then my boss takes over completely and runs a kind of test on you, a little physical activity.

Filling a pause: The doctor creates and fills the space between information instalments with sounds or words indicating the doctor is figuring something out or thinking. In this way, the doctor finds a way to not be interrupted while preparing the next instalment of information. *Filling a pause* is therefore a way to fill spaces in a conversation or discussion and to give the doctor time to think of what to say next.

a. No. Let’s see. It's going to hurt a little bit, put it that way. I hold you.
b. But should you have major problems with this, a relapse, then you can think about putting a toxin like that, botulintoxin(.) You can probably get that done on xx as well. And you are most welcome here again(.) What code is there again then.. anascopy..Uhj. Okay. It was good, it turned out fairly well then.

Generalizing: Associating specific information about the patient’s situation with new information related to the tendencies in the population. This can look like canonical information provision. The specifics about the patient do not have to be in the same utterance and can be implicit.

a. There are always other treatment methods
b. Usually, the majority experience...

Managing responsibility: Making the roles and responsibilities in the interaction or care explicit. Such roles can be in the direction of:

a. giving more knowledge, power, or responsibility to the patient, by asking permission for action or deferring choice
    i. Instead of ”I examine you”, saying ”But let me examine you first”
    ii. Adding to the information message: ”I only give information, I can’t force you to some form of treatment. You decide. I only inform you about how it is”.
b. deferring, expanding, or sharing responsibility with others or to other situations/moments
    i. “We have x-ray meetings and then we discuss it there with the other colleagues, [PT "mhm"] so that I don't have to decide here and now.”

    ii. "And then my colleagues who have seen you before have thought a lot about whether it is not due to alcohol(.)"
c. taking responsibility for a choice or an action
    i. "We just... it was fine that we agree... that I agree with myself [points to herself] [laugh] [LOOK PT] and with you on how to do it"

Opening to the physician colloquialism: The doctor uses a colloquial expression that may be typical of their profession to explain something to the patient. This framing device offers a glimpse into the doctor's professional, but vernacular discourse. It differs *from using precise terms* in the sense that it uses an informal rather than formal register.

a. "You really have what we call the full package for a patient with wear-and-tear damage or wear-and-tear disease in the hip joint. [pat "mhm"]
b. "But... then there is a small but here back at 6 o'clock - you mentioned a small knot that you felt. I also feel a small knot there, and it is bluish, discolored, blue/black"

Rhetorical questioning: The doctor poses a question that does not necessarily project an answer from the patient (there is not a pause after that allows the patient to reply). This framing device usually occurs inside an information giving sequence.

a. Would you consider using medication? It is very important. It is important to prevent you from getting into problems, that's just how I think(.)
b. You know what, then told them that all those examinations were fine (.) [PT "YES"] that they did. (.) [PT "mhm", nods] Nothing wrong with them. [looking at the pc]

Showing competence: With this information framing device, the doctor expresses the source of the information as solid and/or experienced facts. This device is an appeal to the professional experience, knowledge, or role when providing an information message. A specific use of this device is when the doctor traces the history and evolution of a treatment option (see the example in (c)).

a. "This type of surgery is a reasonable standard for us".
b. "And I... yes I think over the years as I have had experience with things I have been more inclined to recommend surgery for your situation"
c. The old surgeons fixed it there with anesthesia and then stretched the sphincter with the result that at the same time they destroyed the sphincter as well. So when the women reached the age of 70, they had leakage problems. So that has been stopped a long time ago. Then there was a period where we made a small incision with a myotomy. But that can also cause slight residual sequelae and a little bad... it has been reported that some percent can get a little bad sphincter then too. [P: "mhm"] And do you have a woman who has had 2-3 somewhat difficult births and the surgeon makes an incision in the sphincter... [P: "mhm"] You only have one sphincter – or two then – and we will don't tamper and damage it too much. So then it was in 95-96, then there were reports in England that nitroglycerin (.) cream on the anal area... it is a chemical way to make the muscle relax [P: "mhm"] And when the wound is healed then you can stop it. [P: "mhm"] And then we are back where it was with a good sphincter and completely sore. It helped 2 out of 3 patients(.) [P: "mhm"] Then there are some new reports later that it may not be so good and you are being fooled a bit by the placebo effect and... [P: "mhm"]

Showing generic practical implications: The doctor provides practical inferences, consequences, repercussions of a message, in the direction of giving indications about what a specific message would practically imply for the patient. Such implications can work for any patient. It leaves for the patient to decide whether it applies to them.

a. You must expect to use crutches for 3-5 days, (.) [P: "YES"] it may be that the knee will be a little tender and swollen and at least painful to bend and such for the first 14 days. [P: "YES"]. <u>So don't add too much work activity</u>.
b. b. Then you will see it become animated or white around here. [INDICATES THE HAND CLOSED] (.) All blood is squeezed away. You don't get a wound to heal with a lack of blood supply. <u>So the treatment (.) goal all along for this has been to get the sphincter to relax (.) [PT "MHM"] Then the blood flow comes back again and it can heal.</u>

Substantiating a medical situation or decision: New information that gives an argument for one medical position or situation, orienting patient to present evidence and a present reason (Why things are as they are/ Why we would do this now.)

a. "There are many good reasons to do this"
b. "Everything you are telling me points in that direction"

Using precise terms: Providing information in a way that displays an effort towards precision (e.g., in terminology, numbers). This information framing device differs from *specifying* as it does not have the sequence from general to specific. Instead, it uses precise, accurate terms to express a concept. It also differs from *clarifying* as it does not provide an explanation or definition for a term, but simply uses the most precise term.

a. Instead of saying "most", saying "90%"
b. Instead of just saying "the instrument" they will use, saying "an instrument called resektoskop"

8. This comes from me as a person

Approximating: The doctor adds hedges to express some uncertainty and that the message is only close or near to the truth or reality; that is, that the borders of the information content are neither completely defined nor clear.

a. Not just "this is something that we have to sort out and see over time" but [continued] "But again, this is something that we have <u>in some way</u> to sort out and see over time."
b. Not just "you get directions here" [continued] But instead "you get directions <u>and all that</u> here".
c. Not just "the bile will flow like a constant stream down the duodenum" [continued] But instead "now the bile will <u>in a sense</u> flow like a constant stream down the duodenum [continued]"

Displaying own professional reasoning: Inserting the professional self into the reasoning about the present situation. This framing device offers an opening into the professional mind of the doctor by personalizing the doctor's relationship to the information provided. It provides a sort of accountability and professional transparency for deliberations expressed in the dialogue. It is

recognizable by the use of a 1st person personal pronoun, followed by verbs indicating a cognitive reflection (I think, I try to understand, I try to clarify, I believe).

a. “What I am trying to imagine is namely..”
b. “This is why I am thinking to..”

Expressing lack or reduced knowledge: Providing information in a way that clearly introduces individual or professional knowledge limits and reveals some degree of personal uncertainty or insecurity about the information message. The doctor takes on his/her shoulders some part of the uncertainty that is intrinsic in medicine.

a. Instead of saying “it is almost impossible to say what type of genetic you have”, saying “I don’t know what type of genetic you have, almost impossible to say”(.)”
b. Instead of saying “And another possibility is that you are in perimenopause”, saying “And another thing is that maybe you are in perimenopause”.

Providing mixed messages: The doctor includes two or more mixed messages that are very different from each other or have contradictory or conflicting meanings, leaving a final impression of ambiguity, unclarity, or inconsistency.

a. “We have this very strong inflammation, but we haven't been able to find that virus. Only a very rare occasion, and therefore we don't think it can explain the whole thing.” [the doctor is both saying that there is a strong inflammation and that the virus potentially explaining can’t explain it]
b. “Because I think that, even if there is nothing quite tangible like that [P:"no"] (.) then there is something, something or other there for which I unfortunately cannot find a very good explanation, but [continued]” [the doctor is both saying that they haven’t find anything and that there is something]

Relating information to personal feelings or emotions: This information framing device offers an opening into the emotional/experiential world of the doctor or of the doctor’s professional group, and it personalizes the doctor’s relationship to the information provided. It is recognizable by the use of a 1st person personal pronoun, singular or plural, followed by verbs or adjectives indicating an emotional state.

a. “What we are most scared of its infection. It happens rarely, and it is something we often manage to come out from, but that it is a very tiring situation for patients and frustrating for us who are responsible for the treatment”
b. “What was a bit silly for us is that if you had started with the nitroglycerin last time, you would have been spared a bit of trouble. But we were so happy that you caught it yourself that you were feeding a child when you might not be so lucky as to give nitroglycerin”

Simulating. The doctor puts him/herself or the patient in the shoes of someone else or in a simulated scenario.

a. So (.) if you were my wife (.) [P: "mhm"] I would probably have put a certain amount of pressure on you because I think caesarean section is the best solution [P: "yes", nods]
b. You could tell him that now I've been to the hospital twice and they can't find anything, shall I really continue with it?- you can do that.

Subjectivizing. The doctor makes the content of the message something that has a subjective sense in that it changes from person to person rather than being objective and a solid fact. This framing device often manifests as "some think that...", contrarily to "some have...". It orients the patient to the degree of possibility that the information could possibly apply to him/her, introducing a sense of uncertainty about the message.

a. No, I would [LIES BACK ON THE CHAIR] recommend it to people if they have problems with it. Because if they don't have problems or think it's cosmetic. But you also understand that there are a lot of people who think this has been troublesome, you know.
b. Many people think it hurts. No, it's not bad, I say. No.

9. **This is directed to you as a unique person**

Translating for the patient: With this specific formulation: "you can say that", the doctor marks that what is coming next is the message said in a way that could be closer to the way a patient might say it (as opposed to in medical terminology).

a. Oh okay. [sighing] No, you can say that based on an overall assessment, we have probably agreed that you should be offered an operation(.)
b. [closed arms, looking at the patient] So you can say that there is very little (.) [pat "mhm"] what I find with you, so it doesn't seem to point in any particular direction. [pat "No"] So I don't want to say like for sure or have like... I feel far from sure of what this is"

Empowering. The doctor encourages and empowers the patient regarding the patient's actions, achievements, or beliefs.

a. "You know your symptoms better than I do. And then you must say it" [also *being directive*]
b. "What was a bit silly for us is that if you had started with the nitroglycerin last time, you would have been spared a bit of trouble. But it was very good that you caught it yourself that you were feeding a child when you might not be so lucky as to give nitroglycerin"

Individualizing. At the opposite of the device "generalizing", in this case the doctor associates and moves from generic information related to the canonical tendencies of the population/situation to specific information about the patient's situation.

a. "When we arrive in week 41-42-43, we still have operating capacity available, so that during the last part of xx or xx, you will have been treated."
b. "What we experience when an ((such an operation against urine leakage helps (.) if there is a slightly sunken uterus(.) That when we put a band around the urethra and one coughs and (.))) [sign with the hands] the uterus and the vagina sinks a little, then there is a small crack in the urethra and then... it stops the leakage. [pat "mhm"] But with you it was perfectly tight and nice (.)"

Reassuring: The doctor uses a formulation in the direction of removing the patient's doubts or worries.

a. “It's good that it has gone so well then, [LAUGH] I have to say that [LOOK MOTHER]. (.) Nothing is better than that”. [the doctor is assuring the patient her actions were good]
b. “If you feel pain, you'll get anesthesia (.) [P: "mhm"] Because you shouldn't feel it (.) That's why you should have some kind of anesthesia (.) [P: "mhm"] Yes (.) So that I can promise you [P: "ok"]” [the doctor is assuring that the patient should have no pain]

Referencing: The doctor adjusts the information message to fit the particular patient’s unique situation. The doctor explicitly refers to something in the patient’s life. This is different from *making the information personal* because it is not moving from general to specific information.

a. **“**And then afterwards we'll fix this so you can go climbing on the mountain again.”
b. “if you don't have any completely crippled anatomy, which you most likely don't have because you haven't never had any such inflammatory conditions and such”

Making the information explicitly personal for the patient: The use of the personal pronoun “you” can be a marker to make the information relevant, specific for the patient. This can happen implicitly throughout the information exchange, but, as the following examples show, there can be more explicit signs of an effort to make the information “personal” for the patient and to stress that the message is something that concerns the patient.

a. Not just “one gets a local anaesthetic”, but “It is also... you get a local anaesthetic, a gel like that, which we inject into the urethra”
b. “And there is... age is not a contraindication for not operating. [pat “no”] And you're quite fit”. [pat "yes"]

Softening: The doctor frames the information in a way that reduces its potential negative weight.

a. Not “since you are old”, but “since you are an adult” (to an older patient)
b. Not “I can’t find an explanation for this”, but “Because I can't find an obviously good explanation for this immediately”

Validating or relating to the patient’s experience: The doctor inserts statements into the information message that relate to the patient’s past, present or potential personal experience.

a. [After the patient having reported problems with the drug] “The Pentasa (.) ehm, we can wait for the time being if you feel it's tiring. Maybe we can get the [flapping the hand on the paper] Imurel treatment going first, (.) and then we can take the other one later.”
b. “So first I will listen to your heart [pat: "mhm"] [touches her own chest], (.) and then I will look at your back [touches her own back] (.) [pat: "mhm"] where you can bend (.) [pat: "mhm"] So if it doesn't go well (.) [pat: "mhm"] you can hope so now [laughter] (.) then we can't do it (.) [pat "ok"] But otherwise we recommend spinal anesthesia.”

Using humor: The doctor designs the information in a way that is comical or amusing, by for example using irony and expressing something that is the opposite of the literal meaning and of what is meant. The expected result of this absurdly incongruity or ludicrous expression, is a laugh.

a. Because otherwise you get to the hospital, (.) and they don’t have the most elegant clothes

b. and if it relapses [changes position on the chair]... it's not so much fun then. [...] Like <u>so much fun as eating the nutritional drink in preparation for the examination</u>.
